# New Drugs, Appliances, and Things Medical

**Published:** 1893-01-07

**Authors:** 


### NEW DRUGS, APPLIANCES, AND THINGS MEDICAL.

[All preparations, appliances, novelties, etc., of which a notice is desired, should be sent for the Editor, to care of The Manager, 140, Strand, London, W.C.]

### DIABETIC FOODS.

Callard, 146, New Bond Street.

We have received samples of the above foods for examination and report. The foods are gluten and almond biscuits and sponge cakes. The manufacturers claim that these former contain less starch than ordinary forms of gluten preparation. We find the starch percentage present a very small one. The biscuits are very nourishing, being made with eggs; have little flavour, and so are not easily tired of; are light, crisp, palatable, and unsweetened; are convenient for the pocket, being small, and not easily broken, and not being like most diabetic foods, domestics and waiters do not recognise them as such. We think that the manufacturers have succeeded in making a standard biscuit for diabetics, and have produced a palatable food. The sponge cakes, which are slightly sweetened with glycerine, are distinctly attractive in appearance and flavour, and will form a welcome change of diet to the many patients who are condemned to the dreary round of non-saccharine food.

### MESSRS. CADBURY'S COCOAS AND CHOCOLATES.

The public did not require the "penny in the slot" machines to educate them to an appreciation of chocolate, but there is no doubt that the sustaining and satisfying qualities of the manufacture have been fully revealed to many a fasting traveller through their means. Many have learnt that it is an excellent plan to be provided with chocolate on any expedition where it is possible that food may not be obtainable when required. Since chocolate is so valuable an article of food, care is not wasted in obtaining the best and purest article possible. Our English manufacturers yield to none in the production of well-made and pure chocolates. Messrs. Cadbury's name, indeed, has become almost a guarantee for pure and wholesome manufactures. They were the first amongst English makers who proved that it was unnecessary to turn to the Continent to procure a starchless cocoa, and those who once have tried their cocoa essence are not likely to leave it in search of a more satisfactory article of foreign manufacture. This preparation of Messrs. Cadbury's is so absolutely pure that the nutty flavour of the cocoa berry is unmistakable. Whilst testifying to the excellence of the chocolates and cocoas which furnish our breakfast table, we cannot refrain from a word of admiration on the really artistic bon-bons produced by this firm. Public taste has become very fastidious in this respect, and the finest sweetmeats are now in general demand amongst the items for dessert. Messrs. Cadbury have risen to the occasion, and various and tasteful combinations are constantly being added to their store of bon-bons. Their chocolate ginger and nugat have been favourites for some time, whilst amongst the latest favourites their chocolate biscuits bid fair to hold a foremost place. These form the most delightful form of intermediate meal that we know. We are all of us practically acquainted with Messrs. Cadbury's excellent productions, and to many of us the method of manufacture adopted would have its interest also. Messrs. Cadbury always extend a courteous welcome to those desiring to visit their Worcestershire manufactory. Those who cannot conveniently do this should read the really interesting history of the manufacture and manufactory entitled "Cocoa: All About It," published by Messrs. Sampson Low, Marston, and Co. If they do this, we venture to predict that they will feel an increased satisfaction in the consumption of Messrs. Cadbury's cocoa and chocolate.

